# The Phosphorylation of Kv1.3: A Modulatory Mechanism for a Multifunctional Ion Channel

**DOI:** 10.3390/cancers15102716

**Published:** 2023-05-11

**Authors:** María Navarro-Pérez, Irene Estadella, Anna Benavente-Garcia, Ruth Orellana-Fernández, Anna Petit, Joan Carles Ferreres, Antonio Felipe

**Affiliations:** 1 Molecular Physiology Laboratory, Departament de Bioquímica i Biomedicina Molecular, Institut de Biomedicina (IBUB), Universitat de Barcelona, Avda. Diagonal 643, 08028 Barcelona, Spain; navarromarya@gmail.com (M.N.-P.); irene.estadella@gmail.com (I.E.); annabenaventeg@gmail.com (A.B.-G.); 2 Departament de Patologia, Hospital de la Santa Creu i Sant Pau, 08041 Barcelona, Spain; rorellana@santpau.cat; 3 Departament de Patologia, Hospital Universitari de Bellvitge, IDIBELL, L’Hospitalet del Llobregat, 08908 Barcelona, Spain; apetit@bellvitgehospital.cat; 4 Servei d’Anatomia Patològica, Parc Taulí Hospital Universitari, Institut d’Investigació i Innovació Parc Taulí (I3PT-CERCA), 08208 Sabadell, Spain; jcferreres@tauli.cat; 5 Departament de Ciències Morfològiques, Universitat Autònoma de Barcelona, 08193 Barcelona, Spain

**Keywords:** K+ channels, cancer, phosphorylation

## Abstract

**Simple Summary:**

The voltage-dependent potassium channel Kv1.3 is a potential target for cancer therapies. This channel exhibits a complex repertoire of physiological functions such as proliferation, activation, insulin sensitivity, nerve action potential and apoptosis among others. Furthermore, the expression and activity of Kv1.3 remodels in various types of tumors. Kv1.3 forms heteroligomeric complexes by association with a number of ancillary proteins that fine-tune the function of the complex. In addition, protein kinase signaling networks are essential for comprehending tumorigenesis. Kv1.3, but also its partners, undergoes phosphorylation-dephosphorylation cycles shaping and remodeling the biology of the channelosome. Therefore, this review compiles protein kinase mechanisms on Kv1.3 to understand regulatory mechanisms during tumor development.

**Abstract:**

The voltage-gated potassium channel Kv1.3 plays a pivotal role in a myriad of biological processes, including cell proliferation, differentiation, and apoptosis. Kv1.3 undergoes fine-tuned regulation, and its altered expression or function correlates with tumorigenesis and cancer progression. Moreover, posttranslational modifications (PTMs), such as phosphorylation, have evolved as rapid switch-like moieties that tightly modulate channel activity. In addition, kinases are promising targets in anticancer therapies. The diverse serine/threonine and tyrosine kinases function on Kv1.3 and the effects of its phosphorylation vary depending on multiple factors. For instance, Kv1.3 regulatory subunits (KCNE4 and Kvβ) can be phosphorylated, increasing the complexity of channel modulation. Scaffold proteins allow the Kv1.3 channelosome and kinase to form protein complexes, thereby favoring the attachment of phosphate groups. This review compiles the network triggers and signaling pathways that culminate in Kv1.3 phosphorylation. Alterations to Kv1.3 expression and its phosphorylation are detailed, emphasizing the importance of this channel as an anticancer target. Overall, further research on Kv1.3 kinase-dependent effects should be addressed to develop effective antineoplastic drugs while minimizing side effects. This promising field encourages basic cancer research while inspiring new therapy development.

## 1. Introduction

Ion channels are crucial components of the cellular machinery that regulates the flow of ions across cell membranes. These proteins participate in the generation and maintenance of the electrical potential, which is vital to many biological processes, including nerve conduction, muscle contraction, and hormone secretion. The wide heterogeneity of ion channels is classified on the basis of channel ion selectivity and the opening mechanism [1]. For example, voltage-gated potassium channels (Kv) respond to fluctuations in the plasma membrane potential by driving potassium down an electrochemical gradient. This highly dynamic process requires fine-tuned regulation; therefore, Kv channels are targets for many posttranslational modifications (PTMs), including phosphorylation [2].

To date, phosphorylation is the most thoroughly studied PTM of Kv channels. Because all Kv channels are susceptible to phosphorylation, the literature contains various examples. Overall, the phosphorylation of Kv channels principally affects protein trafficking, clustering, and gating mechanisms [3]. In addition, targeting phosphorylation by modulating kinase inhibitors constitutes a rapidly developing therapeutic intervention strategy for cancer drug development.

Regarding the Kv1 or Shaker family, tyrosine phosphorylation downregulates Kv1.1, Kv1.2, and Kv1.5 channel activity at the plasma membrane [4,5]. This general effect is mediated by different mechanisms. For instance, the phosphorylation of Kv1.2 destabilizes and increases channel endocytosis [6,7]. On the other hand, serine/threonine phosphorylation of Kv1.2 by protein kinase A (PKA) increases the current independent of channel surface expression [8]. Kv1.4 undergoes similar modulatory effects to reduce the N-type inactivation rate [9].

The phosphorylation of the other Kv families shares similar effects. For instance, whereas PKA-mediated phosphorylation increases the currents in Kv7.1, Kv7.2, and Kv7.3—members of the KCNQ family—tyrosine phosphorylation of these channels downregulates their expression in neurons [10]. Moreover, phosphorylation-related outcomes vary depending on the effector kinase, channel, and targeted residue(s). Hence, in contrast to existing evidence, PKA- [11] and extracellular signal-regulated kinase (ERK-) [12] dependent phosphorylation decreases the current through Kv4.2 [13]. In addition, evidence indicates that PKC phosphorylation can either suppress Kv3.1 [14,15] or increase Kv3.4 currents by eliminating N-type inactivation [16]. In this scenario, some channels, such as Kv2.1, present with high basal phosphorylation levels, and dephosphorylation activates the channel [17].

Overall, the regulation of ion channels via phosphorylation is exceptionally complex and variable. Some Kv channels participate in the proliferation and migration of normal and tumor cells, contributing to metastasis. Thus, some channels, such as voltage-dependent Kv1.3, are highly related to tumorigenesis. Because the altered expression of Kv1.3 correlates with several types of tumors and cancer cells, this protein is widely considered to be an important anticancer target [18,19,20]. Kv1.3 is involved in a plethora of biological events, such as cell proliferation, activation, apoptosis, volume control, and tumorigenesis [21,22]. This review compiles all studies addressing Kv1.3 channel phosphorylation, from the very first observation reported to the most recent advances. In addition, the Kv1.3 channel protein and various related ancillary partners are susceptible to phosphorylation at various sites, which fine-tunes the repertoire of Kv1.3-related biological events [23,24,25,26]. Therefore, the information presented provides a general view of the underlying mechanisms, molecular pathways, and physiological relevance of the Kv1.3 channel to readers.

## 2. Phosphorylation

Phosphorylation constitutes the most common, basic, and important PTM for regulating protein activity and function. In fact, approximately one-third of eukaryotic cell proteins carry one or more phosphoryl groups that are involved in a myriad of regulatory processes. Many of these pathways represent key points in several cell signaling pathways [27]. Interestingly, of the proteins encoded by genes in the human genome, more than 90% can be phosphorylated [28].

Phosphorylation involves the covalent binding of a phosphoryl group transferred from a nucleoside triphosphate (usually ATP) to a serine (Ser), threonine (Thr), or tyrosine (Tyr) residue in a protein. This reaction is catalyzed by protein kinases, which are encoded by approximately 2% of the genes in the total human genome. The attachment of this bulky negatively charged group creates an increased hydrophilic and polar state that might induce considerable changes to the three-dimensional structure of the modified protein. Moreover, the phosphorylated amino acid can react with side chains within the same or adjacent proteins and thus establish protein complexes. Phosphorylation is reversible, with a phosphoryl group removed by a protein phosphatase.

Phosphorylatable Ser, Thr, and Tyr residues are not randomly distributed; in contrast, they are embedded in clear motifs known as consensus sequences within a protein. Hence, the pattern of each consensus sequence confers specific affinity for a unique protein kinase [29,30]. Moreover, these motifs are not merely present in the primary sequence of the protein but might arise when protein folding brings distant residues together.

In addition, regulation via phosphorylation increases the regulatory complexity when a protein carries different consensus sequences recognized by numerous kinases, each of which can differentially modulate target protein activity. In some cases, phosphorylation is hierarchical. Thus, a specific target for kinases may only appear after only a phosphoryl is previously added to a specific residue, which enables the phosphorylation of nearby residues [27,31]. In other cases, specific phosphorylation modulates protein activity to different degrees, or these modifications in combination lead to cumulative effects. In this sense, a proper protein folding, carried by phosphorylable chaperones, assures a correct function [32]. For instance, HSP70 and HSP90 chaperones, which can be phosphorylated, control the Kv1.3 traffic to plasma membrane and mitochondria [33].

Together, these processes exert an extremely refined regulatory effect on protein function. Notably, the removal of a phosphate group is a much less specific process, as reflected by the number of phosphatases (approximately 150) versus the number of kinases (more than 500) encoded in the human genome [28].

In general, reversible covalent modifications are effective regulatory mechanisms, and phosphorylation in particular precisely modulates the most subtle processes [34]. For this reason, phosphorylation is a molecular switch for activating and deactivating many proteins involved in several cell signaling pathways, such as the apoptosis, subcellular trafficking, regulatory metabolism, and proliferation pathways—including those in cancer cells. Therefore, due to its reversibility, versatility, and simplicity, the phosphorylation modification modulates the biological activity of a targeted protein in almost every conceivable way [35].

### Kinases and Phosphatases

As mentioned above, the enzymes critical for adding and removing a phosphate group are kinases and phosphatases, respectively. While kinases show extremely specific and tightly regulated activity, the activity of phosphatases is less specific. Regarding targets, kinases are broadly classified into protein kinases, lipid kinases and carbohydrate kinases. Among these enzymes, protein kinases clearly constitute the largest and most diverse group [28].

The first observation of protein kinase activity dates back to the mid-1950s, almost two decades after the discovery of phosphorylation [35]. Regardless of the time gap between discoveries, the identification of a protein kinase triggered the extensive identification of new kinases throughout the years, and the number of kinases has surpassed 500 [28]. These kinases can be classified on the basis of different characteristics. First, five general families based on their catalytic domain are presented, and then, these families are further classified into nine groups (Table 1). Although this classification was first proposed in 1991, it remains unchanged.

A second method of classification involves the phosphorylation target residue. Notably, serine is the most phosphorylated amino acid (approximately 86.4% of all serine residues can undergo phosphorylation), while threonine and tyrosine phosphorylation are less common modifications (11.8% and 1.8%, respectively) [28]. Hence, serine/threonine kinases constitute the largest family of kinases, which includes AGC, CaMK, GMCC, and some OPKs, while the tyrosine kinase family encompasses the PTK group and some of the OPKs. However, some kinases act on all three residues and are called dual-specific kinases [36]. The aforementioned kinases are the main families in eukaryotic cells; however, prokaryotes and plants also carry histidine-specific and aspartic/glutamic acid-specific kinases, which exert less stable phosphorylation than the serine-, threonine-, and tyrosine-targeted counterparts [28].

Other useful classifications relate to their localization (cytosolic and membrane-spanning protein kinases) or subcellular compartment targeting. Indeed, the compartmentalization of kinases is vital for their proper function and is often controlled by anchoring proteins. The binding of protein kinases to these scaffolding proteins modulates the catalytic activity of the scaffolding proteins, recruiting them to be in close proximity to upstream and downstream effectors. Moreover, one anchoring protein can bind several kinases and phosphatases and thus accurately modulate signal transduction [36].

In contrast, protein phosphatases exhibit functions opposite that of protein kinases. Phosphatases exhibit faster kinetics and less specificity than kinases. Phosphatases have been studied less extensively than kinases because their structures have been more challenging to identify. To date, more than 200 protein phosphatases have been classified into three families: (i) phosphoprotein phosphatases (PPPs), (ii) metallo-dependent protein phosphatase (PPMs), and (iii) protein-tyrosine phosphatases (PTPs). The PPP and PMP families dephosphorylate most pSer and pThr residues and even some pTyr residues in different domain sequences. In general, the PTPs, including the aspartate-based phosphatases, carry the same catalytic domain, with each showing different selectivity. Notably, although less than 2% of all phosphorylation events involve tyrosine, approximately one-half of all protein phosphatases exert their effects on this phosphorylated amino acid [28,37].

## 3. Kv1.3 Phosphorylation

The voltage-gated potassium channel Kv1.3 was first identified in 1985 [38], and shortly thereafter, it was cloned from brain tissue [39,40] and lymphocytes [41]. The expression of Kv1.3 was originally believed to be restricted to the immune and nervous system due to its vital role in excitable cells. However, subsequent studies demonstrated that the channel is ubiquitously expressed and that it participates in a plethora of cellular processes [42]. Similar to kinases, Kv1.3 plays a key role in cell division, proliferation, and apoptosis [21,22]. Moreover, similar to cell cycle regulators in cancer, Kv1.3 has been identified as an important tumorigenic target [43].

Kv1.3 is a delayed rectifier channel, driving a K+ efflux after plasma membrane depolarization. The threshold for activation is approximately −35 mV [44,45]. The channel opens relatively fast, reaching the current peak within 10–15 ms. Sustained depolarization provokes a characteristic C-type inactivation, whereas N-type inactivation is absent. The time constant of inactivation is 250–500 ms, with a slow recovery. Because of this fact, Kv1.3 exhibits a characteristic cumulative inactivation when a train of depolarizing pulses is applied. Kv1.3 kinetics are quite heterogeneous and vary depending on multiple factors, including temperature, blockers, pH, and phosphorylation [44,46].

Kv1.3 is modulated either by accessory subunits or by PTMs. Early reports suggested that the molecular weight of Kv1.3 immunoprecipitated from Jurkat T cells was 65 kDa, in contrast with the protein found in vitro, which is translated into a 58-kDa product. The authors of this study claimed that PTMs caused the discrepancy and pointed to phosphorylation as the main PTM candidate [23].

Although initial studies have described dual PKA- and PKC-dependent regulation of Kv1.3 in Xenopus oocytes [41] and in the human Jurkat T-cell line [47], whether this dual effect was due to direct or indirect phosphorylation was unclear. Metabolic labeling with 32P followed by immunoprecipitation assays demonstrated that Kv1.3 is an endogenous substrate of kinase activity, with serine residues being the main targets [23]. In fact, the cytoplasmic domains of Kv1.3 carry several putative signals for PKA [23], PKC [47], and tyrosine kinase binding [48,49]. Moreover, in vitro phosphorylation study results combined with patch clamp measurements confirmed that both PKA and PKC directly phosphorylated the Kv1.3 channel, triggering its inactivation [23]. Nevertheless, although Kv1.3 can be phosphorylated on different domains, not all of these domains are likely to be equally involved in channel inactivation [50]. Moreover, the study of the effect of kinases on ion channels requires rigorous selection of the cell system. Thus, evidence suggesting that the sensitivity of Kv1.3 to kinases in heterologous systems is less convincing because certain host cell lines cannot recapitulate identical environments or protein factors—such as Kvβ regulatory subunits—that are involved in native cells [51].

### 3.1. Kv1.3 Ser/Thr Kinases

#### 3.1.1. PKA

Although Kv1.3 harbors different putative PKA phosphorylation sites [23], the effect of these sites on the channel has been rarely studied. Notably, in Jurkat T cells, the phosphorylation of Kv1.3 by PKA is quite subtle [23] and depends on prior phosphorylation of the channel by PKC [47]. Moreover, the coordinated and concurrent kinase action of both PKA and PKC exerts an additive effect [52]. Future studies addressing the direct effects of PKA on Kv1.3 may shed light on the general consequences of the phosphorylation of this channel.

#### 3.1.2. PKC

The Kv1.3 amino acid sequence carries several putative PKC-dependent phosphorylation domains. Interestingly, all domains except for one domain have been conserved as mouse, rat, and human orthologs. Rat and human Kv1.3 carries four PKC phosphorylation domains: one in the cytoplasmic loop between the S2 and S3 segments, two between the S4 and S5 cytoplasmic loop, and the one in the C-terminus [53]. One of the sites located in the intracellular loop between the S4 and S5 domains within the Lys-Ala-Ser342-Met-Arg motif has received considerable attention [23]. Notably, this site has been conserved among species, and mutation of the serine reduces the conductance of a single channel by one-half [54]. Therefore, this specific amino acid might control Kv1.3 ion conductivity via the action of a PKC-dependent mechanism [23].

Nevertheless, the general effect of PKC phosphorylation on Kv1.3 might differ depending on the predominant kinase isoform that is modified; its maturation stage, which is related to its expression; and the regulating cofactors involved in the cell type. For instance, untransformed human T lymphocytes express higher levels of PKCβ, while in Jurkat T cells derived from a patient with T-cell leukemia, PKCα is the predominant kinase [55]. Hence, while PKC activators suppress the Kv1.3 current and PKC inhibitors counteract this effect in Jurkat T cells [47] and Xenopus oocytes [41,56], human T lymphocytes show the opposite effects [53].

#### 3.1.3. SGK1

The insulin-like growth factor-1 (IGF-1) signaling pathway involves the activation of several Ser/Thr protein kinases, including the phosphatidylinositol-3 (PI3) kinase [57], phosphoinositide-dependent protein kinase (PDK1) [58], and serum and glucocorticoid-dependent kinase (SGK) 1 [59]. Among these, SGK1 exhibits a stimulating effect on Kv1.3, presumably through the inhibition of the ubiquitin ligase Nedd4-2. Specifically, SGK1 downregulates Nedd4-2, which, in turn, ubiquitinates Kv1.3, causing the endocytosis of the channel. The activation of the IGF-1 pathway has been related to both cell proliferation and apoptosis, in which Kv1.3 also plays an important role [60].

#### 3.1.4. ERK1/2

Adult neurogenesis is thoroughly controlled and restricted to two confined areas of the forebrain: the dentate gyrus of the hippocampal formation [61] and the subventricular zone (SVZ) [62]. Epidermal growth factor (EGF) is a mitogen that triggers signaling that induces neural stem cell proliferation [63]. Specifically, EGF generates a signaling cascade involving serine/threonine and tyrosine phosphorylation that modulates ion channels, including Kv1.3. In fact, EGF modulates Kv1.3 in a dual manner through an unconventional pathway. Notably, EGF reduces channel activity via tyrosine phosphorylation (see below) and triggers channel endocytosis via threonine-dependent phosphorylation, which is mediated by ERK1/2 [64]. Therefore, the combined effects of both EGF-induced channel activation and endocytosis effectively link Kv1.3 to murine neural proliferation [65,66,67].

### 3.2. Kv1.3 Tyr Kinases

Although serine-dependent Kv1.3 phosphorylation has been described [23], the channel shows several potential tyrosine phosphorylation domains, and the literature on Kv1.3 tyrosine phosphorylation has significantly increased recently. The effect of tyrosine phosphorylation on ion channels involves changes in the short and long term, from rapid current variations to slower targeting and protein synthesis [68]. Tyrosine phosphorylation often promotes the metabolic reprogramming of cancer cells. In fact, the tyrosine kinase family includes the largest repertoire of oncoproteins whose upregulation results in cancer [69]. Interestingly, Kv1.3 undergoes remodeling under tyrosine kinase-dependent challenges. Furthermore, Bcl-2 proteins, which are involved in the mitogen-activated protein kinases (MAPK) cascade as either pro- or antiapoptotic proteins, are connected to Kv1.3-related cell proliferation [70]. Hence, different cancer therapies, such as imatinib, gefitinib, and trastuzumab, target tyrosine kinases and have been approved for use and commercialized [69].

Co-expression of Kv1.3 with v-Src (viral sarcoma, oncogenic form of Src) or EGF receptor (EGFR) tyrosine kinases increases channel phosphorylation by eight- and fourfold, respectively, in HEK293 cells [48,71]. A similar effect has been observed in cells treated with the tyrosine phosphatase inhibitor pervanadate. In both cases, the increase in phosphorylation causes Kv1.3 current suppression. Moreover, while pervanadate-dependent phosphorylation does not reverse channel inactivation [48], v-Src co-expression slows C-type inactivation and accelerates Kv1.3 deactivation [71]. Another remarkable difference is that the pervanadate-dependent decrease in the current relies mostly on Tyr449 [48] and at least one tyrosine in the Tyr111-113 triplet of Kv1.3 [72], while v-Src requires the phosphorylation of several tyrosine residues, with Tyr449 and Tyr137 being vital for current suppression. Moreover, in the latter case, diverse combinations of phosphorylated residues exert a differential modulating effect on the channel [71].

#### 3.2.1. MAPK Family

Kv1.3 participates in the proliferation of several cell types by controlling the cell cycle [73]. Kv1.3 favors the G1/S transition mediated through diverse conducting and nonconducting mechanisms [21,74,75]. Among ion-independent mechanisms, Kv1.3 acts as a docking protein that promotes the activation of proliferation signaling cascades. One of these pathways is initiated by platelet-derived growth factor (PDGF) and transduced by MERK/ERK and phospholipase Cγ through the action of various kinases. Kv1.3 carries at least one target residue (Tyr447) whose phosphorylation is MERK/ERK-dependent and necessary for Kv1.3-dependent proliferation. The addition of a phosphoryl group to Tyr447 might create a conformational change in the channel that promotes closed-to-open transitioning, thereby contributing to cell cycle progression [75].

Interestingly, certain elements of the MAPK signaling pathway can be activated downstream of Kv1.3 activation. For instance, Kv1.3 abundance controls the phosphorylation of ERK1/2, a member of the MAPK family that is indirectly activated by either growth factor receptors or tyrosine kinase receptors and thus transduces signals from the plasma membrane to the nucleus. Hence, it is unsurprising that ERK1/2 is intricately involved in cell activation, migration, and proliferation. For this reason, the implications of ERK1/2 dysregulation in cancer have been well documented [69]. In macrophage migration, Kv1.3 plays a pivotal role by upregulating the phosphorylation of ERK1/2 [76].

#### 3.2.2. Src Family

Fas-induced apoptosis, triggered by the Fas receptor activation of the lymphocyte-specific protein tyrosine kinase (lck)—a member of the sarcoma (Src) family—phosphorylates Kv1.3. Interestingly, although the lack of lck completely abolishes tyrosine phosphorylation, channel inhibition is mediated only through an additional mechanism [49]. Shortly after this finding was reported, this additional mechanism was found to be mediated by reactive oxygen species (ROS). Therefore, both Kv1.3 and ROS are important second messengers in Fas-dependent apoptosis, and they both induce protein tyrosine phosphorylation and an increase in lck activity. Interestingly, the oxidation of Kv1.3 by ROS inhibits the current independent of PTK phosphorylation [77,78]. Moreover, a decisive study unified both models by introducing the actions of ceramides, which are lipid metabolites that are released after Fas activation and that activate Src-like tyrosine kinases [79]. Moreover, ceramides can inhibit Kv1.3 in the absence of apoptosis signaling [80]. In Jurkat T cells, the main tyrosine kinase is lck, but the less-expressed fyn kinase can partially overtake the kinase function when the former is downregulated. Hence, the inhibition of Kv1.3 in the lck-deficient Jurkat cell line was masked by fyn compensation [79].

Although lck and Kv1.3 are not directly associated, both bind to Dlgh1 (SAP97), a scaffold protein in the MAGUK family, forming a protein complex in T cells [81]. The proximity of lck and Kv1.3 is important for the lck-mediated Kv1.3 response to hypoxia. When oxygen levels plummet, lck functions as an O2 sensor and, by increasing its own phosphorylation, activates indirect signaling pathways that culminate in the inhibition of Kv1.3 expression and function [82]. Kv1.3 activity is vital for maintaining sustained Ca^2+^ influx during leukocyte activation and proliferation [83]. Thus, hypoxic conditions lead to reduced T-cell activation and attenuated immune responses [82].

Another context in which both Src-family kinases and ROS play independent and dual roles in Kv1.3 is microglial activation. During acute brain insults, such as oxygen/glucose deprivation (OGD), PTKs and K+ channels contribute to microglia activation. In particular, Kv1.3 maintains membrane potential hyperpolarization to promote ROS production and thus combat the threat. However, ROS levels must be tightly regulated, which is realized through a negative feedback mechanism. Thus, ROS promote the phosphorylation of the channel and its subsequent inhibition, thereby limiting the respiratory burst. On the other hand, PTKs in the Src family phosphorylate the channel, leading to the same suppressive effect [78].

Kv1.3-mediated ROS generation is mediated during mitochondrial respiration. An increase in cell respiration relies on both Kv1.3 Tyr447 phosphorylation mediated by ERK1/2 and on a voltage sensor in HEK293 cells. As a result, ROS production increases, generating a proliferative phenotype [84].

#### 3.2.3. Receptor Tyrosine Kinases (RTKs)

##### EGFR

As noted above, EGF efficiently downregulates Kv1.3 channel opening. By activating the EGFR pathway, current suppression relies on the phosphorylation of Tyr479. Similarly, v-Src phosphorylation and EGFR cotransfection change the kinetics of channel inactivation but accelerate C-type inactivation. Both the maximal current decrease and channel inactivation are mediated by separate signaling pathways. Hence, in the case of channel inactivation, in addition to Tyr phosphorylation, Ser/Thr phosphorylation and other mechanisms may be involved [85].

##### Insulin Receptor Kinase

The tyrosine kinase activity of the insulin receptor suppresses Kv1.3 current with no effect on channel inactivation [72,85,86]. The abovementioned dissociation between the decrease in current amplitude and channel inactivation of the channel may explain this outcome. Insulin-dependent phosphorylation is a complex process that involves five tyrosine residues: the Tyr111-113 triplet, Tyr137, and Tyr479 [72,86]. In addition, for this phosphorylation mechanism to be induced, the affected channel must be located in a lipid raft microdomain; otherwise, a defective insulin signaling pathway may result in dysregulated glucose homeostasis [87].

This regulatory effect on the Kv1.3 current is particularly important in the olfactory bulb (OB), where both the insulin tyrosine kinase receptor and Kv1.3 are highly expressed [88,89], with the channel significantly contributing to the outward potassium efflux [86,90]. Notably, sensory deprivation in olfactory bulb neurons increases insulin levels, promoting the phosphorylation and suppression of the Kv1.3 channel, thereby enhancing the sense of smell and odor discrimination. This scenario might be even more complex in cases of dietary restriction, circadian cycle fluctuations, or protein cross-regulation [90,91,92].

In animals with at least one abolished insulin receptor kinase gene allele, the current through Kv1.3 was diminished due to the lower expression of the channel. Although heterozygous animals present with partial preservation of the kinase activity, the modulation of Kv1.3 is strongly susceptible to changes in expression. Interestingly, although Kv1.3 can reciprocally downregulate insulin receptor kinase activity, Kv1.3 knockout mice did not show altered channel expression [91].

In addition to metabolic diseases, such as diabetes mellitus, insulin is an immunomodulator in the central nervous system (CNS) [93]. For instance, insulin enhances locomotor activity by activating the PI3 kinase pathway, triggering Kv1.3 closure [94]. However, the blood–brain barrier impairs the delivery of therapeutic agents. To overcome this obstacle, insulin intranasal delivery (IND) has emerged as a favorable treatment option. Specifically, a single dose of IND increased Kv1.3 phosphorylation in the OB, resulting in current suppression with effects similar to that observed in Kv1.3-knockout mice [92]. Hence, the insulin-induced phosphorylation-dependent modulation of Kv1.3 transforms the channel into a metabolic, endocrine, and neural linker. Depending on the duration of channel exposure to insulin, the activity of Kv1.3 leads to clear firing patterns of the mitral cells in the OB, which act as internal chemical sensors of the metabolic state [95]. Therefore, when IND treatment is prolonged, the phosphorylation of both the insulin receptor kinase and Kv1.3 is dampened, and subsequently, the activity of downstream effectors (AKT/PKB and MAPK/ERK signaling) is mitigated. These signs of early-stage hyperinsulinemia have been observed in mice under chronic IND exposure conditions, and relapsed metabolic and sensory dysregulation was ameliorated by acute treatment [92,96].

##### Trk Family

The tyrosine receptor kinase (Trk) family constitutes another tyrosine kinase-dependent regulator of Kv1.3 in the olfactory bulb. This group of kinases comprises three members: TrkA, TrkB, and TrkC. However, only TrkB carries an active kinase domain. The target neurotrophins are nerve growth factor (NGF), brain-derived neurotrophic factor (BDNF), and neurotrophin 3 (NT3) [68]. Both Kv1.3 and TrkB are co-expressed in specific regions of the OB, such as the internal plexiform layer and in the fibers surrounding glomeruli [97]. TrkB activation by BDNF phosphorylates Kv1.3 with effects similar to those of the insulin receptor but is mediated through a different combination of tyrosine residues (Tyr111-113, Tyr137, and Tyr449) [98]. Moreover, the outcome of Kv1.3 activity depends on the stimulation time and previous odor sensory experience. Thus, upon short and acute insult, the current through Kv1.3 is suppressed without changes in channel kinetics of inactivation or deactivation. Furthermore, in cases of acute stimulation after sensory deprivation, the phosphorylation rate is increased, further decreasing the current. On the other hand, under conditions of chronic stimulation, the current increases, and the inactivation and deactivation kinetics are accelerated [68].

The TrkB receptor increases Kv1.3 expression [97,98]. This upregulation is associated with unique characteristics; for example, TrkB activation is both BDNF-independent but requires basal phosphorylation. Moreover, TrkB stabilizes the channel in the membrane, promoting its release from the endoplasmic reticulum and increasing its half-life. Similar to the insulin receptor kinase, Kv1.3 and TrkB exhibit reciprocal regulatory patterns in vivo, and their levels fluctuate during development. For example, Kv1.3-knockout mice presented with a notable increase in TrkB expression [97]. This dual regulation of protein expression can explain the current increase after prolonged BDNF stimulus; therefore, the expression and subcellular distribution of the channel may vary. Similarly, a current decrease after acute exposition to BDNF may be due to conformational changes or endocytosis [98]. Notably, TrkB modulates the expression of Kv1.5 in a manner opposite to its effect on Kv1.3 [98]. This might indicate a vital role for TrkB in microglial activation, which undergoes a switch from Kv1.5 to Kv1.3 expression. In summary, by downregulating Kv1.5, TrkB attenuates the reduction in the level of the Kv1.3 modulation by forming heterotetramers [99].

Although it is still not clear whether these outcomes are results of direct phosphorylation of the channel or crosstalk between signaling pathways, the causal relationship between phosphorylation and channel activity is evident (Table 2). In addition, this relationship may be caused by either Kv1.3 conformational changes or by a decrease in the number of functional channels in the plasma membrane [48]. Notably, this negative regulatory mechanism is in fact part of a dual inhibitory signaling pathway, as Kv1.3 activity can, in turn, impair protein tyrosine phosphorylation. Although it is unclear whether a decrease in tyrosine phosphorylation is caused by the modulation of tyrosine kinase or phosphatase activity, it functions as a rapid and useful switch-like regulatory mechanism both in vitro and in vivo [100].

## 4. Scaffolding Proteins

Protein phosphorylation requires close proximity between an effector kinase and a target, and scaffolding proteins link several proteins together to optimize signal transduction. Kv1.3, with several molecular domains involved in protein interaction, binds different scaffold proteins. Thus, the channel functions as an adaptor for different proteins through a non-conducting function in addition to its current-conduction action [101]. Sometimes, scaffold proteins disrupt Kv1.3 current suppression [102,103] through a phosphorylation-independent mechanism. In addition, adaptors alter the level of channel expression, which, in turn, controls the levels of kinases such as TrkB [97].

For ion channels, the membrane-associated guanylate kinase (MAGUK) family has been extensively described. The postsynaptic density 95 (PSD-95) protein carries three binding sites: a guanylate kinase (GK), a PDZ, and an SH3 domain. Kv1.3 carries proline-rich sequences and PDZ-binding motifs that can bind the GK-SH3 and PDZ, respectively. Whereas PSD-95-mediated clustering of Kv1.3 relies merely on the PDZ domain, the modulation of the current, kinetics, and inactivation are mediated through the GK-SH3 domain. PSD-95 also binds to the insulin receptor kinase, generating the Kv1.3/insulin receptor kinase/PSD-95 complex. In this context, the SH3 domain in PSD-95 interacts with proline-rich sequences in the insulin receptor kinase catalytic domain, preventing the phosphorylation of Kv1.3 tyrosine. Therefore, in the presence of PSD-95, insulin receptor kinase-induced suppression of the current through Kv1.3 is abolished [103]. Insulin-dependent Kv1.3 tyrosine phosphorylation provokes a PSD-95-mediated association between Kv1.3 and the insulin receptor [103]. Similarly, when insulin is administered intranasally, the scaffolding interactions among the three proteins are enhanced [92]. In summary, PSD-95 binds to Src-family kinases and Kv1.3, favoring the phosphorylation of the channel [78].

SAP97 is another MAGUK family protein that promotes Kv1.3 tyrosine phosphorylation mediated by lck action in T cells. Notably, although fyn can mask lck function in T cells, no interaction between these kinases and SAP97 has been reported [81]. Nonetheless, the possibility of MAGUK protein scaffold formation with other members of the Src family has not yet been ruled out, and more studies on this topic are pending.

As previously described, both Kv1.3 and insulin tyrosine kinases are highly expressed in the OB [88,89], and neurotrophin receptors such as TrkB are also expressed in the OB [68]. This physical location is also enriched with two scaffold proteins: growth factor receptor-binding protein 10 (Grb10) and neuronal Src- and collagen-homology protein (n-Shc) [104]. Grb10 binds and downregulates insulin receptor kinase [105,106], which, in turn, suppresses Kv1.3 current [72,86]. Hence, Grb10 inhibits Kv1.3 downregulation. On the other hand, Shc binds TrkB (a BDNF receptor), promoting the phosphorylation and subsequent suppression of Kv1.3 activity [101]. Sometimes, an adaptor protein needs to be phosphorylated by a kinase to be activated. For instance, Grb10 and n-Shc are Src substrates, similar to Kv1.3. However, although Grb10 reduces the Src-dependent Kv1.3 tyrosine phosphorylation rate by competing for a binding site of the kinase, n-Shc produces an increase in the phosphorylation rate of the channel but does not affect the current [101,102]. Grb10 and n-Shc exert opposite effects on Kv1.3 expression: n-Shc increases Kv1.3 expression, and Grb10 reduces it, presumably through Nedd4-2-mediated endocytosis. This modulation is reciprocal, as Kv1.3 reduces the levels of both adaptors. Despite the antagonistic effects of Grb10 and n-Shc on Kv1.3, they both prevent BDNF-induced current suppression in the OB [101].

When certain receptor tyrosine kinases (RTKs), such as insulin receptor and TrkB, are activated, they are phosphorylated, creating new and specific recognition sites for scaffolding proteins. Therefore, the pattern of tyrosine phosphorylation may generate another filter that, depending on the recruited adaptor, a specific kinase may phosphorylate Kv1.3 with a particular outcome [98].

Finally, the abovementioned tyrosine kinase Src targets caveolin-1 (CAV1), which, in turn, binds Kv1.3 through a caveolin-binding domain in the N-terminus of the channel [22,107]. Although CAV1 has been extensively described to play an oncogenic role—in fact, it was the first described Src substrate correlated with cell transformation—CAV1 also shows tumor suppressor activity [107]. This antagonistic role depends on the cancer type and clinical stage. CAV1 targets Kv1.3 in lipid rafts at the plasma membrane and in mitochondria [22,108]. The extent to which Src-mediated phosphorylation of CAV1 modifies Kv1.3-related tumor progression is unknown. Nevertheless, a mitochondrial Kv1.3-CAV1 axis has been suggested to be a major factor in Kv1.3-mediated apoptosis and the chemotherapy resistance of cancer cells [21,22,109].

## 5. Phosphorylation of Regulatory Subunits

Kv channels form heterooligomeric structures (Kvα subunits) with peripheral accessory subunits. These auxiliary proteins modulate several Kvα morphogenic, structural, and physiological properties, as well as channel subcellular localization. Overall, the main accessory proteins comprise Kvβ subunits and KCNEs as well as, ancillary BK channel subunits, namely K+ channel-interacting proteins (KChips) and dipeptidyl aminopeptidase-like proteins (DPPL), specifically for Kv4 channels [110]. Because this review focuses on the Kv1.3 channel, only the Kvβ and KCNE regulatory subunits are discussed.

### 5.1. Kvβ

Kvβ subunits were the first Kvα accessory proteins to be characterized. They are encoded by three different genes (KCNAB1, KCNAB2, and KCNAB3) that produce six different splicing variants [111,112]. Kvβ is formed by an exceptionally highly conserved protein core with a variable NH2 terminal domain [110]. Notably, this variable domain harbors several putative PKA-, PKC-, and CSK2-phosphorylation sites [26,113,114], which are critical for the differential kinase-mediated regulation of each Kvβ isoform [113]. Evidence indicates that Kvβs are phosphorylated by PKA and PKC [24,26], which represents a “modulation of the modulator” mechanism with respect to Kv1.3. Altering a regulatory subunit property leads to intricately refined modulation of Kvα subunits. Nevertheless, studies focusing on Kvβ phosphorylation are rare.

The first observation of Kvβ phosphorylation was made serendipitously by scientists studying PKA-dependent Kv1.3 phosphorylation [23]. Subsequent studies demonstrated that PKA phosphorylation of Kvβ subunits leads to physiological consequences for the Kvα protein in heterologous systems. Sometimes, Kvβ differential modulation after PKA phosphorylation is caused by Kvα subunit phosphorylation, which regulates the Kvα/β interaction [115]. Hence, both Kvα and Kvβ subunits are kinase targets, and the degree of their respective phosphorylation determines the outcome of their functional interactions [115,116].

Kvβ1 accentuates the inactivation of Kvα with respect to the current, playing an essential role in cardiac cells. Kvβ1.3 can be phosphorylated by either PKA or PKC [51,113,117,118]. PKA phosphorylation alleviates Kvβ1.3-dependent Kvα inactivation. Interestingly, this effect relies on only one residue (Ser24) in the variable N-terminus of Kvβ1.3 [113]. The effect of PKC on Kvβ1.3 encompasses electrophysiological and trafficking outcomes. When PKC is inhibited, the electrophysiological properties and pharmacological effects that Kvβ1.3 exerts on the Kvα subunits are abrogated, restoring a Kvα-only activity pattern [118]. Moreover, the recycling of the channel and the subunit to the plasma membrane is impaired [117]. Overall, PKC plays a considerable role in the trafficking of Kvβ1.3 and the Kvα subunit to the plasma membrane, promoting rapid Kvβ1.3-induced inactivation of the channel [117,118].

Kvβ2 lacks a ball-and-chain domain that would enable its regulation of Kvα inactivation. However, Kvβ2 is the most abundant and widely expressed Kvβ subunit, participating in the processing and targeting of Kvα subunits. In addition, Kvβ2 presents aldo-keto reductase activity by binding NADPH with high affinity [112]. Although Kvβ2 carries ten putative PKC-phosphorylation sites and only one site for PKA phosphorylation, Kvβ2 phosphorylation accounts for most of the PKA activity in vitro. However, PKC phosphorylation prevails in the rat brain due to the expression of adaptor proteins [114]. Kvβ2 is mainly phosphorylated by PKC in the N-terminus and close to the NADPH-binding site, which probably mediates the coupling process [26]. Although Kvβs are phosphorylated in heterologous systems, Kvβ2 does not modify channel susceptibility to PKA or PKC regulation [51].

Again, adaptor proteins physically link the Kvβ subunit and the kinase. Notably, ZIP1 and ZIP2 proteins, which bind both Kvβ2 and PKC-θ, facilitate phosphorylation [119].

### 5.2. KCNEs

The KCNE family of ancillary subunits includes five members with one transmembrane domain. Each subunit is encoded by a single gene, whose mutations have been associated with several diseases. KCNEs participate in the trafficking and gating properties of Kv channels, although outcomes differ by the stoichiometry, specificity for the Kv subunit, and PTMs [110].

All five KCNEs carry multiple putative phosphorylation sites for many kinases, but direct phosphorylation has seldom been reported to date. However, one example is the PKC-mediated Ser82 phosphorylation of KCNE3, which leads to changes in the electrophysiological pattern of the Kvα current [120,121]. On the other hand, the PKC-mediated phosphorylation of KCNE1 at Ser102 leads to the internalization of the Kvα/KCNE1 complex [122,123,124] and downregulation of Kvα currents [125]. Many studies have highlighted the requirement of a KCNE subunit for the modulatory effect of the phosphorylated Kvα subunit. For instance, KCNQ1 is phosphorylated by PKA independently of KCNE1 or KCNE2, but the C-terminus of these ancillary subunits is vital for current regulation [126,127,128].

The mere expression of KCNEs is essential for controlling physiological phosphorylation levels under some conditions. In fact, the lack of KCNE2 or KCNE4 in mice induced fluctuations in the phosphorylation rate of certain components of signaling cascades in response to ischemia/reperfusion injury (IRI) [25,129]. On the one hand, KCNE2 deletion increased the basal phosphorylation of several kinases (ERK, p38 MAPK, JNK, AKT, and GSK-3β). However, after IRI, the phosphorylation of these elements was impaired [129]. KCNE4, which is an effective regulatory subunit of Kv1.3 [130], exerted similar effects. In this case, the lack of KCNE4 increased only the GSK-3β basal phosphorylation level and impaired ERK phosphorylation after IRI [25].

KCNE subunits also form complexes with adaptor proteins to reduce the distance between the kinase and Kvα subunit. For instance, Yotiao (AKAP9) is a scaffold protein that binds KCNEs, Kvα subunits, and PKA [126,128].

## 6. Functional Consequences of Kv1.3 Phosphorylation

As mentioned above, the human genome encodes more than 500 kinases, and more than 90% of human proteins are phosphorylated. Hence, protein phosphorylation constitutes a widespread regulatory mechanism whose dysregulation might affect several types of cells to different degrees. Notably, approximately 50% of altered human kinases have been reported to correlate with a disease. In fact, many alterations in phosphorylation-dependent kinase functions result in cancer [69]. For this reason, interest in the development of pharmaceutical compounds that target dysregulated kinases has been increasing [131].

One well-documented disease characterized by the dysregulation in protein phosphorylation is acquired immunodeficiency syndrome (AIDS), which is caused by human immunodeficiency virus (HIV). In the case of Kv1.3, the HIV-induced outcomes vary depending on the cell type. In lymphocytes, the course of infection is characterized by an initial decrease in CD4 T-cell number and function [132]. The viral gp120 membrane glycoprotein plays a pivotal role in spreading the infection, as it is detached from infected lymphocytes and crosslinks the CD4 receptor on healthy T cells. Once attached to a new host lymphocyte, gp120 alters the activity of TCR downstream elements, such as lck [133,134] and PKC [135], resulting in increased phosphorylation of Kv1.3 and subsequent current suppression. After Kv1.3 downregulation, lymphocytes are depolarized, and the Ca^2+^ flux required for T-cell activation is impaired [136].

However, HIV exerts an opposite effect on Kv1.3 phosphorylation in oligodendrocytes. The viral protein Tat interacts with Kv1.3, interrupting channel regulation mediated via phosphorylation. Therefore, the channel is more active, which results in increased oligodendrocyte injury [137].

Interestingly, tyrosine kinases are most highly expressed in the nervous system. For this reason, many types of kinase dysfunction can trigger CNS diseases. As mentioned above, the insulin receptor is highly expressed in the OB, playing an important regulatory role mediated through Kv1.3 action [89]. Thus, dysfunction to the insulin signaling pathway, such as the disruption in patients with diabetes mellitus, alters the function of Kv1.3 and leads to olfactory dysregulation. Briefly, diabetic patients present reduced insulin receptor kinase activity, which results in hampered Kv1.3 phosphorylation and a subsequent increased current. Considering that Kv1.3 produces most of the current in mitral cells in the OB, their electrical signaling is notably impaired, which diminishes olfactory function [91]. In this context, the search for new pharmacological targets has been at the epicenter of many recent studies. As mentioned above, insulin intranasal delivery (IND) has led to treatments that can bypass the blood–brain barrier. Therefore, immunomodulation via insulin can be exogenously controlled through a minimally invasive method [92]. However, dosing should be monitored in a timely manner because prolonged exposure time causes opposite effects, as explained above. Unfortunately, the wide and varied targets of phosphorylation as well as their unclear outcomes represent obstacles in the form of undesired side effects. Therefore, future therapeutic treatments need to avoid targets that function as signaling nodes, and efforts therefore need to be directed to less interconnected targets in the phosphorylation cascade.

As illustrated in the previous sections of this review, both Kv1.3 and kinases are key regulators of the cell cycle, proliferation, and apoptosis [21,22,69]. Therefore, dysregulation of either Kv1.3 or the kinases that phosphorylate the channel is closely related to carcinogenic outcomes [69]. Therefore, many anticancer drugs target either Kv1.3 kinases or the channel. For instance, the commercialized drug gefitinib selectively inhibits the kinase EGFR, which has been previously described to downregulate and modulate the inactivation of Kv1.3 through different phosphorylation sites [85]. Although it is unclear whether this compound could exert a direct effect on Kv1.3, the channel—and putatively some other Kv channels—is involved in gefitinib resistance of some cancer cells. Hence, a treatment combining gefitinib and Kv blockers has been suggested [138].

Several epidemiologic studies have shown that soy-based diets correlate with a lower cancer risk. The reason is related to the soy isoflavone genistein, which effectively blocks protein tyrosine kinases. Therefore, genistein affects Kv1.3 function via two separate pathways. First, it inhibits the tyrosine kinases that phosphorylate the channel. However, genistein also inhibits Kv1.3 in a rapid kinase-independent manner, which might involve a direct interaction. This compound is a potential therapeutic target for both cancer prevention and as an adjunct therapy. However, further basic and clinical studies are needed before the definitive approval of genistein for use in clinics [71,139,140].

Altogether, Kv1.3 phosphorylation is undoubtedly subject to many therapeutic approaches. Nonetheless, the entangled phosphorylation pathways and their heterogeneous outcomes have hindered the development of putative pharmacological drugs.

## 7. Conclusions

**Figure 1:**
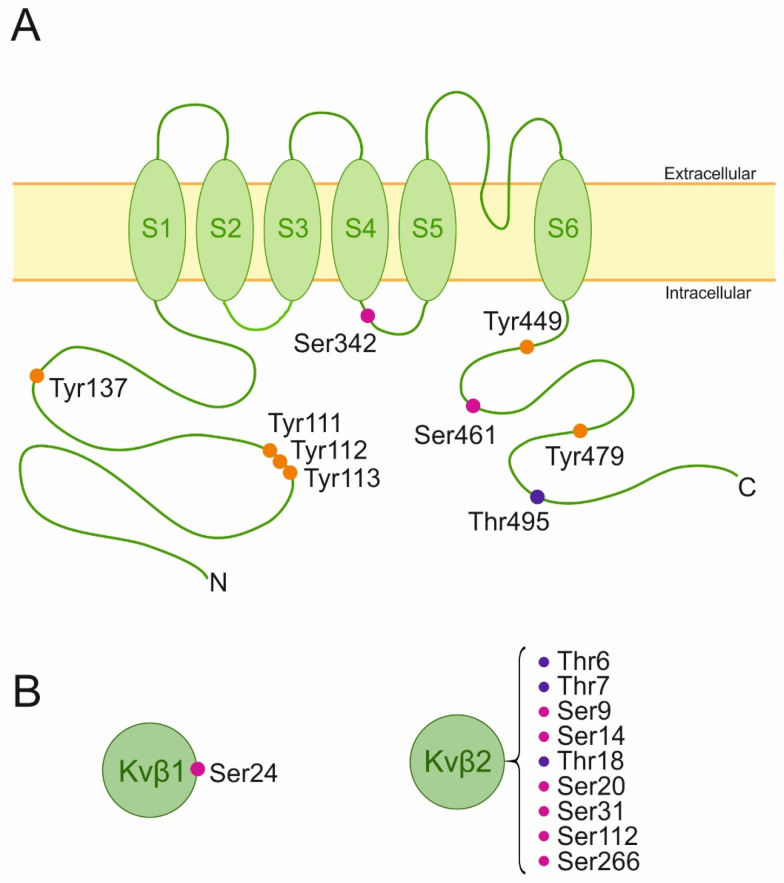
Schematic representation of Kv1.3 membrane topology and Kv1.3 beta ancillary subunits highlighting putative phosphorylation residues. (**A**) The channel presents six transmembrane domains (S1–S6) and intracellular N and C terminal domains. (**B**) Cytosolic Kvβ subunits. Phosphorylated amino acids are highlighted with the three-letter code and colored dots. Magenta (serine), purple (threonine), and orange (tyrosine) [141].

Kv1.3, which is involved in cell proliferation and tumor progression, carries several consensus sequences that bind different serine/threonine and tyrosine kinases (Figure 1). In addition, abnormal phosphorylation signaling is strongly associated with tumorigenesis. Concomitantly, kinases are potential targets for drug development against cancer. Therefore, compounds targeting phosphorylation pathways, some of which are in clinical trials, represent promising possibilities for cancer therapy. However, a phosphorylation outcome is not black or white, but rather exhibits a wide range of gray tones. The variability of the resulting effects relies on multiple factors such as the target amino acid, the kinase, the cell type, the maturation stage, and ancillary and scaffold proteins.

Thus, an a priori basic PTM generates intricate regulatory mechanisms, complicating the study of these modifications. Paradoxically, the study of PTMs requires exhaustive research because many altered phosphorylation events correlate with disease. Moreover, the ubiquitous expression of Kv1.3 may lead to the extrapolation of these alterations to the whole body, especially the immune and nervous systems. Therefore, it is of vital importance to increase our understanding of Kv1.3 phosphorylation so that particular attention can be devoted to the design of specific anticancer therapeutic treatments.

## Figures and Tables

**Table 1:** General classification of eukaryotic protein kinases. Attending to the target amino acid, protein kinases are classified as serine/threonine and tyrosine kinases. Regarding the catalytic domain, serine/threonine kinases are grouped into the AGC group (protein kinases A, G, and C), Ca^2+^/calmodulin kinase (CaMK), Cell Kinase 1 (CK1), GMGC group (after CDK, MAPK, GSK, and CDK-like kinases), STE (sterile) kinases, tyrosine-like kinases, and other protein kinases (OPK). Tyrosine kinases are divided into cytoplasmic or receptor (RTK) tyrosine kinases. Each group is formed by different families and presents general traits [34].

Serine/Threonine Kinases			
Group	Families	Principal Members	General Traits
AGC group	14	PKA, PKG, PKC, SGK, AKT/PKB, PDK1, PKN/PRK, RSK, NDR, MAST, YANK, DMPK, GRK, SGK494	Activated by cyclic nucleotides (PKA by cAMP and PKG by cGMP), regulated by Ca^2+^, diacylglycerol, or phosphatidylserine (PKC), among others. The “PIF pocket” serves as kinase regulation site and therapeutic target.
Ca^2+^/calmodulin kinase (CaMK)	17	CaMK1, CaMK2, PSK, DAPK, MLCK, CASK, PHK, DCAMKL, MAPKAPK, CAMKL, TSSK, PIM, Trbl, PKD, RAD53, Trio, VACAMKL	Binding of Ca^2+^/CaM complex results in release of the auto-inhibitory domain from the N-terminal kinase domain. Classified into substrate-specific or multifunctional kinases. Key activators of transcription factors.
Cell Kinase 1 (CK1)	3	CK1, VRK, TTBK	Regulators of signal transduction pathways related to proliferation, cell differentiation, chromosome segregation, and circadian rhythms.
CMGC group	8	GSKs, MAPK, CDKs, CTD, DYRK, SRPK, CLK, RCK	Essential modulators of the cell cycle (CDKs), glycogen metabolism (GSKs), diverse signaling cascades involved in proliferation, differentiation, neural plasticity (MAPK), and the spliceosomal complex (CLK), among many others.
STE	3	Ste7/MAP2K, Ste11/MAP3K, Ste20/MAP4K	Also known as MAP2K, MEK, or MKKs, important activators of MAPK family.
Tyrosine kinase-like (TKL)	7	IRAK, MLK, RIPK, STKR, RAF, LRRK, LISK	Most diverse group with sequence similarity to tyrosine kinases but lacking specific motifs.
Other Protein Kinases (OPK)	37	PLK, Aur, CAMKK, TLK, ULK, IKK, NAK, NEK, TTK, MOS, TOPK, PEK, WEE, CDC7, NKF1, CK2, BUB, Bud32, Haspin, IRE, NKF4, NKF2, NKF3, NKF5, NRBP, Wnt, SLOB, SCY1, VPS15, TBCK…	Extremely diverse group of Ser/Thr and dual kinases.
		**Tyrosine Kinases**	
Group	Families	Principal Members	General Traits
Cytoplasmic tyrosine kinases	32	ABL, ACK, CSK, FAK, FES, FRK, JAK, SRC, SYK, TEC…	Critical regulators of the immune system by controlling cell growth, proliferation, differentiation, migration, and apoptosis.
Receptor tyrosine kinases (RTKs)	20	EGFR/ErbB, IR, PDGFR, VEGFR, FGFR, CCKR, NGFR, HGFR, EphR, AXLR, TIER, RYKR, DDRR, RETR, ROSR, LTKR, RORR, MuSKR, LMRR, other RTKs	They contain a transmembrane domain and a highly conserved intracellular C-terminal region with kinase domains.

**Table 2:** Summary of Kv1.3 phosphorylation residues and physiological relevance. As shown, only two serine/threonine kinases have been reported to phosphorylate specific amino acids of the channel. Kv1.3 is phosphorylated in diverse tyrosine residues with different outcomes depending on the kinase. PKC: Protein Kinase C, ERK1/2: Extracellular signal-Related Kinases, v-Src: viral sarcoma, EGFR: Epidermal Growth Factor Receptor, PDGFR: Platelet-Derived Growth Factor Receptor, IRK: Insulin Receptor Kinase, TrkB: Tropomyosin Receptor Kinase B.

Serine/Threonine Kinases		
Kinase/Trigger	Residues	Outcome
PKC [23]	Ser 342	Modulation of Kv1.3 ion conductivity
ERK1/2 [59,70]	Ser 461 Thr 495	Kv1.3-induced cell proliferation EGF-dependent Kv1.3 endocytosis
**Tyrosine Kinases**		
Kinase/Trigger	Residues	Outcome
v-Src [66]	Tyr 111-113 Tyr 137 Tyr 449 Tyr 479	Current suppression and fast deactivation (Tyr 137, Tyr 449) Slow C-type inactivation (more than 3 Tyr)
EGFR [80]	Tyr 479	Decreased peak current independent of inactivation
PDGFR-ERK1/2 [70]	Tyr 449	Kv1.3 induced mitochondrial respiration and cell proliferation
IRK [67]	Tyr 111-113 Tyr 137 Tyr 479	Insulin-mediated current suppression
TrkB [93]	Tyr 111-113 Tyr 137 Tyr449	BDNF-induced current decrease (Tyr 111-113, Tyr 137, Tyr 449) Modulation of BDNF-dependent inactivation (Tyr 137)
Pervanadate [67]	Tyr 111-113 Tyr 449	Pervanadate-dependent current suppression
